# Understanding effects of armed conflict on health outcomes: the case of Nepal

**DOI:** 10.1186/1752-1505-4-20

**Published:** 2010-12-01

**Authors:** Bhimsen Devkota, Edwin R van Teijlingen

**Affiliations:** 1 Section of Population Health, School of Medicine and Dentistry, AB 25, 2ZD, University of Aberdeen, Scotland, UK; 2 Associate Professor, Tribhuvan University, Kathmandu, Nepal; 3 School of Health & Social Care, Bournemouth University, Dorset BH1 3LT, Bournemouth, UK & Visiting Professor, Manmohan Memorial Institute of Health Sciences, Nepal

## Abstract

**Objective:**

There is abundance of literature on adverse effects of conflict on the health of the population. In contrast to this, sporadic data in Nepal claim improvements in most of the health indicators during the decade-long armed conflict (1996-2006). However, systematic information to support or reject this claim is scant. This study reviews Nepal's key health indicators before and after the violent conflict and explores the possible factors facilitating the progress.

**Methods:**

A secondary analysis has been conducted of two demographic health surveys-Nepal Family Health Survey (NFHS) 1996 and Nepal Demographic and Health Survey (NDHS) 2006; the latter was supplemented by a study carried out by the Nepal Health Research Council in 2006.

**Results:**

The data show Nepal has made progress in 16 out of 19 health indicators which are part of the Millennium Development Goals whilst three indicators have remained static. Our analysis suggests a number of conflict and non-conflict factors which may have led to this success.

**Conclusion:**

The lessons learnt from Nepal could be replicable elsewhere in conflict and post-conflict environments. A nationwide large-scale empirical study is needed to further assess the determinants of Nepal's success in the health sector at a time the country experienced a decade of armed conflict.

## Background

Violent conflicts pose a challenge to human civilisations, human health and health systems [1-3]. Epidemiological studies indicate that war ranks among the top-ten causes of death worldwide [4-6]. Populations affected by armed conflict experience severe public health consequences mediated by population displacement, food scarcity, and the collapse of basic health services, which together often give rise to complex humanitarian emergencies [7,8]. Conflict has both direct and indirect effects on people's health and on the overall health system [8]. Armed conflicts can also cause the displacement of people and an increase in infectious diseases [2,9].

Nepal recently emerged from a decade-long violent conflict (1996 to 2006). This violent conflict had an effect on both the population's health and the health care system[10-12].It led to over 13,000 fatalities[13], the disappearance of at least 1,200 people [10,14], the disablement of thousands of people, and the internal displacement of many more [14,15]. Over 1,000 health posts in rural areas were destroyed [16], more than a dozen health workers had been killed and many others were harassed, kidnapped, threatened and prosecuted by the warring factions [14,17,18]. The conflict aggravated the already poor health services as one third of Nepal's health centres is in rural areas (where some of the fighting was heaviest) and often operates without health staff [19-21]. Torture and sexual-abuse related to insurgency were also prominent [11,22,23], and the conflict also hindered health programmes implemented by non-governmental organisations [24,25].

The Maoist rebels put restrictions on field staff mobility and both the security forces and rebels tried to stop public gatherings focused on health-related awareness. Furthermore, the Maoists objected to the implementation of the Community Drug Programme (CDP) by opposing the minimal fees associated with it.

Nepal and 146 other countries adopted the Millennium Development Goals (MDGs) in 2000 [26]. The MDGs are eight targets to be achieved by 2015 to overcome the key global development challenges (Table 1). Hence MDGs are a yardstick against which we can measure progress made by the member countries (or lack thereof) in terms of health and development indicators. Three out of eight goals (i.e. MGD 4-6) relate directly to health, and health is an important contributor to several other MDGs.

**Table 1:** Millennium Development Goals (MDGs)

1	Eradicate Extreme Poverty & Hunger
2	Achieve Universal Primary Education
3	Promote Gender Equality & Empower Women
4	Reduce Child Mortality
5	Improve Maternal Health
6	Combat HIV/AIDS, Malaria & Other Diseases
7	Ensure Environmental Sustainability
8	Develop a Global Partnership for Development

Amidst the civil war, Nepal appeared to have made improvements in its human development index, life expectancy and child and maternal health indicators [18,21,27]. Some of the publicly available datasets suggest that Nepal has made considerable progress on certain key health indicators, however for few other indicators the progress seems to have stagnated. In recognition of its progress on reduction in maternal and child mortality rate and improvement on other health indicators, the US (United States) Government recently commended Nepal under its Global Health Initiative Programme. Nepal is only one of eight countries receiving this award. Nepal's progress seems puzzling as it contradicts our common understanding that civil conflict is an impediment for improving the health services. It raises a question whether the progress was real, and if so, what could have contributed to achieve this progress? The possible hypotheses could be that Nepal's violent conflict: i) worsened health indicators; ii) improved health indicators; or iii) had a mixed effect, i.e. improvement in some and stagnation or deterioration in other indicators. This paper analyses Nepal's main health indicators before and after the conflict and offers some possible explanations for the observed changes.

## Methods

This paper is based on secondary analysis, in which "data collected by one researcher are re-analysed by another investigator usually to test new research hypotheses" [28]. Thus secondary analysis uses data which have already been collected and the research question might, or might not, have formed part of the remit of the original study design. In this paper we draw upon data from three main sources: i) demographic health surveys 1996, which were called Nepal Family Health Survey (NFHS) in 1996 [29] and Nepal Demographic Health Survey (NDHS) in 2006[30]; ii) a study led by the first author under the auspicious of Nepal Health Research Council (NHRC) in 2006 [27]; and iii) data from the Ministry of Health and Population (MOHP) and similar sources. The NFHS 1996 used household questionnaire and women interviews while the NDHS 2006 used household interviews and separate interviews with women and men. This paper compares health indicators based on the household interviews (particularly demographic characteristics, water, sanitation, nutritional status of children) and women interviews (e.g. education, marriage, childbirth, family planning, fertility, maternity care, immunisation, awareness of HIV/AIDS) in order to address gender biases whilst comparing the 1996 and 2006 data.

The NFHS 1996 and NDHS 2006 both used multistage systematic sampling; each covered all three ecological regions (i.e. mountain, hill and terai) and all the five development regions of Nepal (i.e. Eastern, Central, Western, Mid-western and Far-western regions). The NFHS 1996 covered 8,429 women aged 15-49, while the NDHS 2006 covered 10,793 women aged 15-49 and 4,397 men aged 15-59. Both surveys were conducted by the same two organisations-Macro International (technical support) and New Era ( a local research firm) under the aegis of the Department of Health Services. These conditions permit comparison of the NFHS 1996 and NDHS 2006 data. The sampling and data collection methods used in these two studies allow us to make a valid comparison for pre-and post-conflict juxtaposition. The analysis focuses on women (See notes in Table 2), since the 1996 study did not include interviews with men, while the coverage of all three eco-regions and cross-section of five development regions ensures the whole country is covered. Though the Maoist violence started in the western part of the country in 1996, it had spread all over Nepal by 2001, hence it was not possible to define 'conflict' and 'non-conflict' areas and disaggregate the data to make comparisons between these two areas.

**Table 2:** Main health indicators at the beginning (1996) and end of the conflict (2006)

MDG Goal	Health Indicators*,**	1996 (NFHS)	2006 (NDHS)	Difference	OR	95% CI		NHRC 2006	MDG Target 2015
						Lower	Upper		
GOAL1 Eradicate extreme poverty & hunger	1. Percent of stunted children under 3 (height/age)	56	42	14	1.756	1.003	3.077	Na	30
	2. Percentage of undernourished children under 3 wasting (wt/height)	11	15	-4	0.700	0.3.45	1.6109	Na	25
	3. Underweight children under 3 (weight for age)	42	35	7	1.344	0.759	2.381	Na	29
GOAL 4 Reduce child mortality	4.Neonatal mortality rate/1,000 live births	50	33	17	2.030	1.145	3.598	Na	16
	5.Infant mortality rate/1,000 live births	79	48	31	3.915	2.108	7.283	Na	34
	6.Under 5 child mortality rate/1,000 live births	118	61	57	2.059	1.491	2.843	Na	54
Intermediate Indicator	7. DPT 3 immunisation coverage %	76	87	11	0.472	0.225	0.993	93	100
	8. Measles vaccine coverage %	57	85	28	0.233	0.118	0.460	91	90
GOAL 5 Improve maternal health	9.Maternal mortality ratio/100,000 live births	539	281	258	2.991	2.484	3.602	Na	134
	10. Total fertility rate	4.6	3.1	1.5	1.333	0.298	5.959	Na	2.4
Intermediate Indicator	11.Current use of any modern method of contraception among currently married women 15-49 years %	26	51	25	0.355	0.196	0.641	53	67
	12. ANC visit %	26	75	49	0.276	0.152	0.501	68	NI
	13.TT shots during pregnancy(2 or more) %	33	63	30	0.289	0.161	0.517	81	NI
	14.Delivery attended by skilled personnel %	10	19	9	0.473	0.208	0.078	43	60
GOAL 6 Combat HIV/AIDS, Malaria and other diseases	15.Tuberculosis prevalence rate/100,000 population	310҂	280	30	1.107	0.942	1.302	Na	Halt and reverse
	16.Malaria prevalence rate/100,000 population	52҂	25	25	2.080	1.291	3.352	Na	Halt and reverse
	17.Prevalence of HIV in age group 15-49	Na	0.5	-	-	-	-	Na	Halt and reverse
GOAL 7 Ensure environmental sustainability	18.Access to drinking water(improved source)	33	82	49	0.108	0.055	0.208	Na	68*
	19. Access to sanitation %	20	42	22	0.412	0.223	0.760	Na	53

Note: Na = Not available, NI = Not included, OR = Odds Ratio, CI = Confidence Interval

҂ = The figures are for 2000 as no data was available for 1996

҂҂ = Universal access target is 100%

* Indicators 1-3 and 18 and 19 are based on household questionnaire data,** Indicators 4-14 and 17 are based on women questionnaire data,

** Indicators 15 and 16 are based on MOHP data presented in a national MDG workshop in Kathmandu on February 10, 2010.

There is a possibility that problems occurred during the data collection of the various surveys. The insecurity due to conflict made the survey data collection less reliable [31] since (a) parts of the country was not under Government control; and (b) Census enumerators might have been afraid to approach people whom they believed to be Maoist sympathisers as Census enumerators were working for the Government. Some of this may also have occurred during the data collection for the studies used in our secondary analysis.

The study conducted by the NHRC in 2006 covered 800 women with children under the age of two, 40 health service providers, 145 key informants, 104 exit clients at the service outlets and 400 focused group discussion participants from across 10 districts representing all five regions of Nepal [27]. The methods and tools of this study does not seem compatible to the NFHS 1996 and NDHS 2006. Moreover, the sample size of the NHRC study is relatively small. The results of the NHRC study however give a comparative picture on 6 out of 19 indicators included in Table 2. It offers data for supplementation to the NDHS 2006. The qualitative data from the NHRC study (2006) are used to supplement the analysis of the changes over time (where available and appropriate).

## Results

Table 2 presents the key health indicators before the start of the violent conflict in 1996 and immediately after denouncement of violence by the Maoists in 2006.The data are presented into two sub-headings; health outcomes demonstrating improvement and health outcomes that remained stagnant or even worse during the decade-long conflict.

### Health outcomes demonstrating improvement

The data suggest that there has been progress in the reduction of stunting and underweight among children under three years (MDG 1), by 14% (OR 1.756, CI 1.003-3.077) and 7% ( OR 1.334, CI 0.759-2.381) respectively. In case of MDG 4, the infant and child mortality rates have dropped by 31% and 57% respectively and the coverage of childhood vaccines (intermediate indicators) increased over the years. Both DHS surveys show that coverage of DPT 3 and measles vaccines increased by 11% and 28% respectively, however the pace of progress appears to be slower. The coverage of DPT 3 and measles as shown by the NHRC study seems little higher (i.e. 93% and 91% respectively) than the NDHS 2006. It suggests likelihood of achieving the MDG targets by 2015.

Similarly, the progress on two indicators of MDG 5 shows that achieving overall MDG 5 appears to be possible. The goal of reduction in maternal deaths is likely to be achieved as it reduced from 539 to 281(OR 2.991, CI 2.484-3.602). The total fertility rate has dropped from 4.6 to 3.1 over the decade (OR 1.333, CI 0.298-5.959). Out of the four intermediate goals related to MDG 5, three goals (i.e. increase in modern contraceptive use, ANC visits and receiving Tetanus Toxoid vaccines (TT) by pregnant women are likely to be achieved. Between 1996 and 2006 contraceptive use increased by 25%, ANC visits by 49% and the TT uptake by 30%. The MDG 6 reversal and halting of tuberculosis and malaria could also be achieved as likelihood of the former seems to be 1.1 times higher (OR 1.107, CI 0.942-1.302), while the latter is two times higher(OR 2.080,CI 1.291-3.352) in 2006 compared to the NFHS 1996.

The HIV prevalence in the 15-49 year age group was not available in NFHS 1996 which remained at 0.5% in 2005[32]. Table 2 suggests two targets under MDG 7 (access to drinking water and sanitation) are possible to achieve. The proportion of population with access to drinking water increased by 49% despite the conflict while increase in access to sanitation stood at 22%.

Further indicators add the notion that Nepal is making progress in its health status such as the decrease in unmet need for family planning (31% in 1996, 25% in 2006) and the improvement in overall life expectancy from 56.5 years in 1996 to 63.3 years in 2006[33].

### Health outcomes that remained stagnant/worse during the conflict

Despite the progress in most health outcomes in Table 2 Nepal's goal of reducing the proportion of undernourished children was reversed by 4% over the period of violent conflict. The prevalence of under-nutrition however appears to be lower than the MDG 2015 target (25%). Similarly the pace of reduction of the neonatal mortality rate (MDG 4) of 17% over the past decade suggests that reaching the neonatal mortality target for 2015 is going to be a serious challenge. Moreover, one of the indicators of the MDG 5-delivery attendance by skilled personnel increased by 9% against the reference year, which needs to be increased by 49% in order to achieve the MDG target of 60% in 2015.

## Discussion

From the point of view of the impact of the conflict, the data available from the two DHSs suggest more of a positive than of a negative impact on the health outcomes. The comparative data on 19 MDG-related indicators show that 16 out of 19 indicators had improved to such a level that MDG would be likely to be achieved by 2015. While two indicators-reductions in neonatal mortality and improvement in skilled attendance at birth had increased at a slower pace, hence the related MDGs are unlikely to be achieved. One indicator, the percentage of undernourished children under three years old worsened in 2006 compared to the reference year 1996. Most of these findings on the trend of progress are compatible to the trends of health indicators shown in the MDG Progress Report published by Nepal's National Planning Commission in 2010 [32]. According to this report "Nepal is **likely **to meet the targets on reducing under five mortality by two-thirds, reduce the maternal mortality ratio by three quarters, halt and reverse the spread of HIV/AIDS, halt and reverse the incidence of malaria and other major diseases and halve proportion of population without sustainable access to improved water source. It is **potentially likely **to meet the targets on achieving universal access to treatment for HIV/AIDS for all those who need it. However, the report reiterates that Nepal is **unlikely **to meet the targets of achieving universal access to reproductive health and halving proportion of population without sustainable access to improved sanitation"[32].

Contrary to evidence from other conflicts [8,34-37] as well as from Nepal [38-40] of a negative impact of conflict on the health of populations, we found that in Nepal progress has been made in most health indicators. There does not appear much literature on what made it possible to achieve such progress despite a decade-long armed conflict. The discussion below explores the key drivers contributing to the better than expected changes in people's health status in a period of civil unrest and armed violence.

The first possible explanation is that Nepal's warring sides, in particular the former rebels, did not purposively disrupt the delivery of health services [41]. The health sector appeared to have been less susceptible to the violence. Besides few sporadic incidents, the overall political outlook of the rebels towards the health programmes and health workers was positive. Special national campaigns such as the National Immunisation Day for polio and measles immunisation, bi-annual vitamin supplementation and family planning camps were not much affected [16]. The key informant district health officers from Far-western districts expressed that the Maoist insurgents did not interrupt health activities in their districts.

*Though the conflict had limited people's mobility for seeking our services particularly during transportation strikes (bandhs), they (Maoists) did not stop us from providing our services to the people *(District Health Officer ID 5, Mid-western Region).

A second explanation is that the former rebels put pressure on the health care providers in their 'base areas' or the contested areas to attend regularly at clinics in order to ensure consistent drug supplies and treatment [42]. As a result, the government was under pressure to supply appropriate health staff and supplies. In spite of the security threat, 78% of staff positions in hospital, 75% in primary health care centres (PHCCs), 96% in health posts and 90% in sub-health posts were filled during the conflict [27].

Thirdly, conflict created an environment for improved coordination amongst the key actors: the MOHP, donors, civil society and the community representatives. One Local Development Officer's remark reflected this:

*We have improved coordination between the district government and health representatives. We conduct regular meeting and discuss issues of local development, including those related to the health sector*. (Key Informant ID 11)

The example of improved coordination despite the conflict in Nepal was also found during conflicts in East Timor [43] and Mozambique [37] where improved coordination amongst the key stakeholders helped increase utilisation of health services by the local population. In Nepal, it encouraged inclusive, people-based and transparent humanitarian programmes at the local level. Exemption of user fees to poor and disadvantaged populations and provision of citizen charters (*agarics adapter*) at service outlets could be taken as examples [27]. It also recognised the role of civil society and the local community groups in these health development activities.

Though the service guidelines have special provisions for poor and disadvantaged patients, there were problems however in defining them when it came to implementation [27,44]. One participant in a focused group discussion (FGD) said:

*The service guideline directs us to providing free health services to the DAG (disadvantaged groups) and poor people but there are no clear definitions who they are. The decision depends on the discretion of the doctor attending the patient. (*FGD 2, District ID 7)

Fourthly, building on the lessons from the protracted conflict, Nepal's public health system adopted a number of health improvement approaches and programmes. Some of the key policies focused on disadvantaged groups including *dalits*, women, disabled and elderly people, whilst helping to increase coverage of the health programmes in more remote and underserved areas. The policies also included the establishment of emergency funds and community drugs schemes and handing over the government ownership of the health facilities to the local communities [27].

Fifthly, Nepal strived to maintain a visible, sustained and adequate provision of health services at all levels from the centre to the community. There has been a substantial increase in the number of health care institutions, from 1,098 in 1991 to 4,552 in 2007/2008 [45]. The Government health facilities, such as health posts, sub-health posts, primary health care centres and outreach clinics provided basic community-based services, mostly free of charge. Nepal implemented many popular programmes such as the community-based integrated management of childhood diseases (CB-IMCI); community-based newborn care package(CB-NCB), community drug programme (CDP); direct observation treatment system (DOTS) for treatment of tuberculosis; HIV and AIDS prevention and control programmes; rural water supply and sanitation programme (RWSSP) and a food security programme. These initiatives helped increase access to and utilisation of the available health services [27,32].

Sixthly, there was a functional community support system including the Health Facility Management Committees, mothers groups, Female Community Health Volunteers (FCHVs) and Traditional Birth Attendants (TBAs) for the mobilisation of local communities. One study showed that one-thirds of women were member of local women's groups, and that 43% members of the health facility management committees were from lower socio-economic groups such as Janajatis and *dalits *[27]. However, motivation and performance of these groups were often questionable in terms of their voluntariness as opposed to their desire for economic incentives, including the coping strategy in the context of the political conflict [46].

Seventhly, the UN (United Nations) and various international non-governmental organisations (INGOs) contributed for increasing the coverage and effectiveness of the health services in Nepal. They implemented conflict-sensitive development programmes whilst keeping a low profile [47]. Nonetheless, in the absence of clear government policy and elected representatives, coordination between the government, development partners and the community people appeared to be poor [27].

Eighthly, development of infrastructures such as road, health facilities, schools, electricity, and communication might have contributed to the positive changes. One study found that despite the frequent transportation blocks due to strikes, more women living near main roads sought care from maternal health services [44]. Additional evidence is that access to health services increased over the years, for example travel time fell 50 times between 1995/96 and 2003/4 [21]. The NHRC study shows 83% women and 71% of service users reported having access to a health facility within 30 minutes' walk, with a further 16% of women and 14% of service users had reached within one hour on foot. Similarly, of the total service-users interviewed 51% in the terai, 45% in the hill area and 4% in the mountain districts had access to a road. However, focus groups with women from a remote district highlighted a lack of access to health services still existed.

*People from here should either travel on horseback for four days, or fly to Pokhara (regional headquarter) via aeroplane to get treatment in a hospital*. (FGD 1, District ID 13)

Increase in access to education and communication could have supported positive changes in health outcomes. During the decade of 1996-2006, adult literacy increased from 34% in 1996 to 79% in 2006 [29,30]. The primary school enrollment rate increased from 57% to 73%. In 1996, only 7% of all households had a radio and television, which increased to 28% in 2006 [33].

Ninthly, Nepal achieved a steady economic growth and substantial reduction in poverty. Between 1995/96 and 2005/6, the percentage of the population living below the poverty line (US$1/day) decreased from 42% to 31%, and the absolute poverty dropped by one percentage points per year over the past couple of years. This somehow seems to contradict the economic explanation on the causation of conflict that underdevelopment and poverty fuels conflict [48-50]. However, a 2005 regional poverty profile shows that Nepal has varying regional deprivation levels. During 2003-2004, Kathmandu had the lowest level of poverty (3%) while the other urban and rural areas had higher poverty levels i.e. 9.6% and 34.6% respectively [51]. The Nepal Living Standard Survey (NLSS II), 2003/2004 also reveals discrepancies in the distribution of poverty by development regions. It is lowest in the Central Development Region (27%) and highest in the Mid-western Development Region (45%), which is considered as the epicentre of the Maoist insurgency [52].

Economic inequality was reported between (a) the centre and the periphery; (b) the 'haves and have-nots; (c) different castes; and (e) people with different levels of education. For instance, in Kathmandu the average gross domestic product (GDP) was almost four times higher than that of some rural regions [52].

The increase in government's health sector budget, though only a small percentage change, might have helped towards achieving these health outcomes. The share of health sector budget increased from 5.99% in 1995/96 to 6.41% in 2005/2006 [32]. Moreover, the share of foreign aid of total government expenditure increased from 17.96% (2001/2002) to 19.88% in 2005/2006 and its contribution in Nepal's development expenditure increased from 58.07% to 74.45% [32]. Similarly, the share of foreign aid to GDP in the same period increased from 3.13% to 3.37% [32].These inputs would have contributed to the positive changes in the health indicators.

## Conclusion

In spite of the violent conflict, Nepal made progress in 16 out of 19 health indicators over the period 1996-2006. The indicators of universal access to reproductive health, halving proportion of population without sustainable access to improved sanitation and proportion of underweight children has remained stagnant. We have outlined nine possible factors that help explain this phenomenon of seemingly improved health outcomes in a time of war. It is, of course, very likely that a combination of these nine factors interacted to create the positive environment in Nepal, despite, or perhaps because of its internal conflict.

The lessons from Nepal are that in order to ensure functional delivery of health services and improvement in health outcomes during conflict, the warring sides should adopt a strategy of coexistence and the international community should continue and increase their support to strengthen the health sector with a principle of 'do-no-harm' and impartiality and the government should implement conflict-sensitive measures and improve coordination amongst the key actors. Moreover, the overall national economic and social context should be conducive to bridging divides, and finally the government should work to fulfill its commitment towards the national policies and programmes and international instruments. It is equally important to reform the health services by building on Nepal's experience and consider the positive transformations that can occur as a result of conflict.

As this was the first comparative study that examined the health outcomes before and after the conflict and presented available evidences to explore the reasons for the positive changes, this paper provides general trend of health indicators overtime. Future studies should try to differentiate between conflict affected and peaceful areas and look at the conflict attributes that generate positive and negative consequences for the health services. Perhaps a little more focus is needed on the positive aspects as most of the studies conducted elsewhere portray negative consequences of conflict and ignore the transformation that occurs as a result of conflict.

## Competing interests

The authors declare that they have no competing interests.

## Authors' contributions

BD analysed the data and prepared draft of the paper.

EVT finalised the manuscript of the paper.

Both the authors have read and approved the final manuscript.
